# Transgenic Mouse Overexpressing Spermine Oxidase in Cerebrocortical Neurons: Astrocyte Dysfunction and Susceptibility to Epileptic Seizures

**DOI:** 10.3390/biom12020204

**Published:** 2022-01-25

**Authors:** Manuela Marcoli, Chiara Cervetto, Sarah Amato, Cristian Fiorucci, Guido Maura, Paolo Mariottini, Manuela Cervelli

**Affiliations:** 1 Department of Pharmacy, Section of Pharmacology and Toxicology, University of Genova, Viale Cembrano 4, 16148 Genoa, Italy; amato@difar.unige.it (S.A.); maura@difar.unige.it (G.M.); 2 Interuniversity Center for the Promotion of the 3Rs Principles in Teaching and Research (Centro 3R), Lucio Lazzarino 1, 56122 Pisa, Italy; 3 Department of Science, University of Rome “Roma Tre”, Viale Marconi 446, 00146 Rome, Italy; cristian.fiorucci@uniroma3.it (C.F.); paolo.mariottini@uniroma3.it (P.M.); 4 Neurodevelopment, Neurogenetics and Molecular Neurobiology Unit, IRCCS Fondazione Santa Lucia, Via del Fosso di Fiorano 64, 00143 Rome, Italy

**Keywords:** Polyamines, SMOX, glutamate excitotoxicity, reactive astrocytosis, epilepsy, transgenic mouse model

## Abstract

Polyamines are organic polycations ubiquitously present in living cells. Polyamines are involved in many cellular processes, and their content in mammalian cells is tightly controlled. Among their function, these molecules modulate the activity of several ion channels. Spermine oxidase, specifically oxidized spermine, is a neuromodulator of several types of ion channel and ionotropic glutamate receptors, and its deregulated activity has been linked to several brain pathologies, including epilepsy. The Dach-SMOX mouse line was generated using a Cre/loxP-based recombination approach to study the complex and critical functions carried out by spermine oxidase and spermine in the mammalian brain. This mouse genetic model overexpresses spermine oxidase in the neocortex and is a chronic model of excitotoxic/oxidative injury and neuron vulnerability to oxidative stress and excitotoxic, since its phenotype revealed to be more susceptible to different acute oxidative insults. In this review, the molecular mechanisms underlined the Dach-SMOX phenotype, linked to reactive astrocytosis, neuron loss, chronic oxidative and excitotoxic stress, and susceptibility to seizures have been discussed in detail. The Dach-SMOX mouse model overexpressing SMOX may help in shedding lights on the susceptibility to epileptic seizures, possibly helping to understand the mechanisms underlying epileptogenesis in vulnerable individuals and contributing to provide new molecular mechanism targets to search for novel antiepileptic drugs.

## 1. Polyamines Metabolism

**Figure 1:**
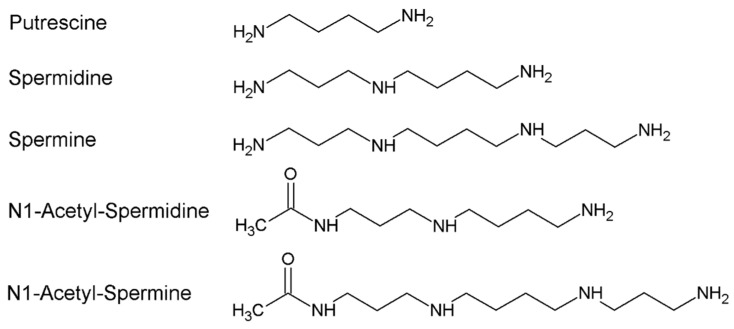
Polyamines and their acetyl derivatives.

Polyamines (PAs) are organic polycations ubiquitously present in living cells. The main PAs in mammalian cells include putrescine (Put), spermidine (Spd), and spermine (Spm), and their acetylated forms, N^1^-acetylspermidine and N^1^-acetylspermine (Figure 1).

**Figure 2:**
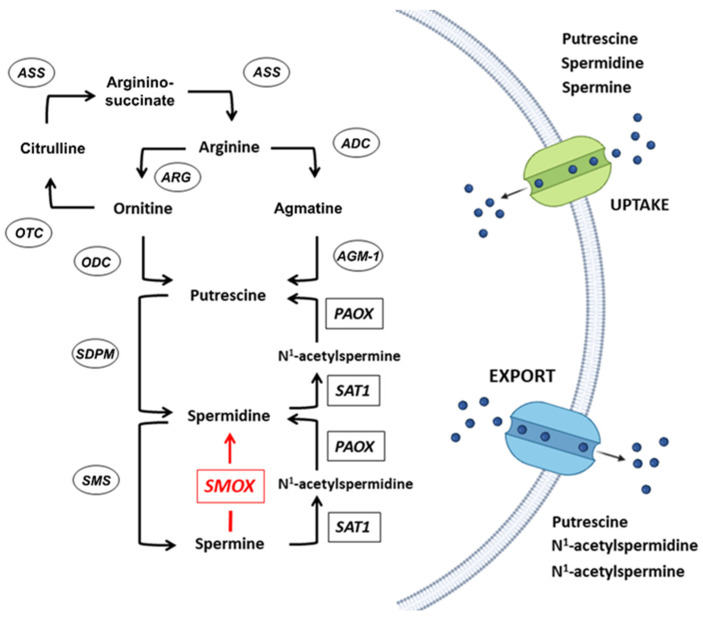
Polyamine’s metabolism. Enzymes involved in PA biosynthesis and catabolism are encircled and boxed, respectively. ADC, arginine decarboxylase; AGM-1, agmatinase; ARG, arginase; ASS, arginino-succinate synthase; ODC, ornithine decarboxylase; OTC, ornithine transcarbamylase; PAOX, N^1^-acetylpolyamine oxidase; SAT1, spermidine/spermine N^1^-acetyltransferase; SMS, spermine synthase; SPDS, spermidine synthase. Spermine oxidase (SMOX) is highlighted in red and is overexpressed in the DACH-SMOX transgenic mice model, (ARG), (AGM1), (ADC), (OTC).

The PA content in mammalian cells is tightly regulated [1,2,3] and it has been linked to important cellular roles. Polyamines are involved in the synthesis of proteins and nucleic acid and in the maintenance of their structure, in the regulation of the activity of ion channels, in cell proliferation, differentiation, and apoptosis, as well as in protection from oxidative damage [1,2,3]. Altered PA cellular levels have been reported in several pathological conditions of the Central Nervous System (CNS). One of the best examples is the low level of Spm observed in the Snyder–Robinson syndrome, an intellectual disability disorder with movement disorder and seizures, due to a rare mutation of the Spm synthase gene in the X-chromosome [4]. Alteration in the Pas’ synthesis and metabolism have been reported to be correlated with suicidal behavior [5]. Multiple symptoms including neurological abnormalities are also reported in rodent models of altered PA synthesis and catabolism [6,7]. Notably, PAs have been suggested to counteract cognitive impairment in animal models by activating autophagy and mitochondrial function, and high dietary Spd intake correlated with lower risk for cognitive impairment in humans. Polyamine biosynthesis is carried out by the action of four enzymes: S-adenosylmethionine decarboxylase enzyme (AdoMetDC), ornithine decarboxylase enzyme (ODC), spermine synthase (SMS), and spermidine synthase (SPDS) [8]. While a PA catabolic pathway is dependent on the activity of three enzymes: N^1^-acetylpolyamine oxidase (PAOX), spermidine/spermine N^1^-acetyltransferase (SAT1), and spermine oxidase (SMOX) [7,9,10,11]. Figure 2 depicts a schematic representation of the PA metabolism.

## 2. The SMOX Overexpressing Mouse: An Animal Model of Chronic Spm Catabolism Activation

**Figure 3:**
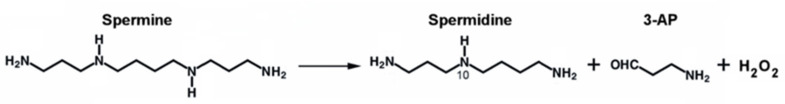
Spermine oxidase enzymatic reaction. The substrate spermine is oxidized to produce spermidine, 3-aminopropanal (3-AP), and hydrogen peroxide (H_2_O_2_).

The enzyme SMOX specifically recognizes Spm as a substrate to produce Spd, with the production of hydrogen peroxide (H_2_O_2_) and 3-aminopropanal (3-AP) (Figure 3) [12,13,14].

**Figure 4:**
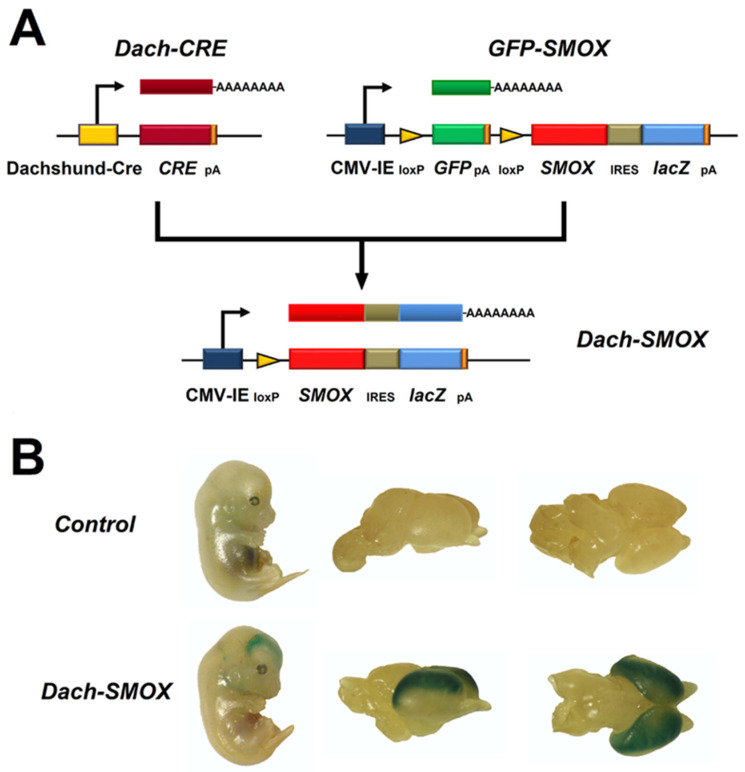
Scheme of Dach-SMOX mouse generation. (**A**) CRE recombination scheme between Dach-CRE and GFP-SMOX to obtain Dach-SMOX mouse lines. (**B**) Left-side, LacZ staining of wholemount at E12.5 mouse developmental stage of Dach-SMOX and control embryos; right-side, LacZ staining of brains at E14.5 mouse developmental stage of Dach-SMOX and control embryos.

The SMOX enzyme is expressed in various tissues, mainly in brain and skeletal muscle, and regulates the Spm/Spd ratio to keep the cellular PA content balanced [7,15]. Apart from its role in the basal PA metabolism, the SMOX substrate Spm has an important function in the brain, since intracellular Spm is also a neuromodulator responsible for intrinsic gating and rectification of strong inward rectifier K^+^ channels (Kir) by directly plugging the ion channel pore [16,17,18]. Moreover, the intracellular level of Spm, by plugging the receptor channel pore, can also cause inward rectification of some subtypes of alpha-amino-3-hydroxy-5-methyl-4-isoxazole- propionic acid (AMPA) and kainate Ca^2+^-permeable receptors in the CNS [19,20]. Furthermore, extracellular Spm has multiple effects at the N-methyl-D-aspartate (NMDA) subtype of glutamate receptors, increasing the intensity of NMDA receptor currents and voltage-dependent blocks [16,17,18]. The oxidation product of SMOX Spd is also a neuromodulator, but less potent than Spm since it binds the same channel receptors with much less affinity [17]. Several works have highlighted new roles for Spd that protect from age-induced memory impairment acting directly at synapses by means of an autophagy-dependent homeostatic regulation [21] or enhancing eEF5/EIF5A hypusination, cerebral mitochondrial function, and cognition in aging Drosophila melanogaster and mice [22]. It has been demonstrated that Spd induces autophagy in several model systems, including rodent tissues and cultured human cells [23,24,25]. The integrity of the autophagic system has been suggested to be crucial for the Spd-mediated protection from age-associated presynaptic active zone changes and increase in the active zone scaffold components in D. melanogaster [26]. Furthermore, SMOX activity not only controls Spm/Spd cellular ratio, but it is also a source of cellular redox alteration by producing H_2_O_2_, a reactive oxygen species (ROS). Hydrogen peroxide can also modulate brain Long-Term Potentiation in a dose-dependent manner, but an excessive increase of it can result in learning and memory impairment [27]. Spermine oxidation by SMOX is also responsible for secondary tissue damage, due to the generation of 3-AP, which spontaneously converts into acrolein [11,28]. Noteworthy, the production of acrolein in the injured brain is a further source of inflammation and apoptotic cell death; in line with this, a decrease in Spm content and an increase in plasma protein conjugated-acrolein (PC-Acro) could be considered a good marker for brain infarction [28]. Furthermore, in stroke patients, the high levels of SMOX, PAOX, and PC-Acro in plasma correlates with the stroke size [29,30,31,32,33,34]. A mouse model conditionally overexpressing SMOX in the neocortical neurons with a CD1 background, formerly JoSMOrec, now DACH-SMOX, has been engineered (Figure 4) [12,35].

The Dach-SMOX mouse line is a chronic model of excitotoxic/oxidative injury and neuron vulnerability to oxidative stress and excitotoxic insults [36,37,38]. Notably, the mice revealed to be a chronic model of increased susceptibility to seizures that might help to understand neuron vulnerability to insult as well as epileptogenesis. This mouse genetic line provides a potential model to be further exploited, in addition to simple acute epilepsy animal models, for new pharmacological approaches to cure epilepsy. The results obtained from the study on SMOX overexpressing mice are of relevance also in the scenario of the increasingly recognized importance of astrocytes in brain function [39,40,41], which is now shifting from a neurocentric to a neuro-astrocentric view [42]. In fact, chronic overexpression of SMOX in cortical neurons of Dach-SMOX mice severely affects their astrocyte morphology and function, and heavily influences cerebrocortical synapse functioning [43]. The findings support roles for endogenous PAs in maintaining neuron–astrocyte cross-talk and in neuroprotection [44], and that an imbalance of PA synthesis and flux can alter the neuron–glial communication in the brain [16,44,45]. Consistently, accumulating evidence indicates that PAs are synthesized in neurons, released from neurons into the extracellular space, and preferentially accumulated in glial cells [46] from glial cells, they can then be secreted back to neurons [45]. Indeed, in SMOX-overexpressing mouse changes of PA metabolism and H_2_O_2_ overproduction co-occurring in cerebrocortical neurons seemingly to affects astrocytes, and in turn, neurons. In particular, reactive astrocytosis and neuron loss are the main effects of the chronic activation of Spm catabolism in cerebrocortical neurons in Dach-SMOX mice, together with chronic oxidative stress and excitotoxicity [36,37,38]. The main changes and their molecular mechanisms in the Dach-SMOX line are highlighted in the following paragraphs.

## 3. Reactive Astrocytosis

The relevance of neuron-astrocyte networks to the control of signal transmission and the regulation of brain function is widely recognized [39,40,47,48]. Astrocytes provide neurons with energy substrates, nutrients and neurotransmitter precursors, structural support around synapses, and buffering of the excess of K^+^ and neurotransmitters in the extracellular space [49,50,51,52]. Astrocytes regulate extracellular glutamate concentration by balancing its uptake through the glutamate transporters EAAT1 and EAAT2, and its release and uptake through the antiporter cystine-glutamate exchanger xc^−^ [53,54]. Furthermore, astrocytes and, in particular, the astrocytes processes, can release glutamate in a Ca^2+^-dependent vesicular or Ca^2+^-independent ways [36,37,55,56,57,58,59,60,61,62,63,64]. The perisynaptic astrocytic processes (PAPs), which envelop synapses and are primarily involved in astrocyte-neuron communication at tripartite synapses [56,61], display rapid movements and Ca^2+^ elevations in response to neuronal activity [65], and regulate coverage of synapses, synapse plasticity, and the interstitial space volume [66]. In response to brain injury, astrocytes undergo morphological, molecular, and functional remodeling, also known as the so-called reactive astrocytosis [50,67]. The contribution of reactive astrocytes to CNS diseases and repair is matter of debate, and both detrimental and neuroprotective actions have been attributed to reactive astrocytes [68]. Reactive astrogliosis in the cerebral cortex of Dach-SMOX mice is indicated by an increased number of astrocytes and morphological cellular changes consisting in hypertrophy and wide ramification [12,36]. Consistent with the presence of reactive astrocytes and a neuroinflammation condition [69], a relative abundance of astrocyte processes versus nerve terminals was observed in Dach-SMOX mice, with the increase in GFAP-positive particles, and a reduction of synaptophysin-positive particles [36], together with increased levels of the astroglial markers ezrin and vimentin [43]. Indeed, vimentin is a potential marker for reactive astrocytes [67,68] of relevance in the control of the function of astrocytes and astrocyte processes in reactive astrocytosis [70]. Furthermore, an increase in ezrin, a protein preferentially localized in the fine PAPs unsheathing synapses [71], specifically indicates changes of these fine processes. This might have consequences on the synapse function, as PAPs are involved in glia-synaptic interactions, and ezrin is required for PAPs motility and regulation of synapse coverage [72]. Indeed, ezrin participation in both neuroprotective and neurotoxic activities of the reactive astrocyte processes is a matter of intense study [71]. Altogether, these findings point out a typical remodeling of reactive astrocytes in response to chronic activation of PA catabolism. Astrocytes in the cerebral cortex of Dach-SMOX mice may become reactive, possibly responding to overproduction of H_2_O_2_ due to SMOX overexpression, and/or as a consequence of neuron impairment. In fact, oxidative stress and inflammation are significant factors promoting reactive astrocytosis [73], and at the same time, activated astrocytes are capable of generating ROS [74]. In addition, synaptic neuronal activity is crucial to maintain healthy astrocytes by trophic signaling that can regulate astrocyte function [65]. It can be hypothesized that PAs are among these trophic factors produced in neurons, then released and accumulated in glial cells [16,44,45]. In turn, reactive astrocytes may have detrimental effects on neurons, contributing to the dysregulation of synapse functioning [43]. The processes of reactive astrocytes in Dach-SMOX mice undergo the following modifications that might impact on glutamatergic synapse function: reduced Spm content, expression of AMPA GluA2-lacking receptors linked to Ca^2+^ entry and activation of glutamate release, increased xc^−^ transporter function and increased glutamate in-out transport, reduced expression of EAAT1 and EAAT2.

## 4. Reduced Spm Content in Reactive Astrocyte Processes

No change of Spm content in the cerebral cortex of SMOX over-expressing mice was measured [12], consistent with the tight regulation of PA homeostasis. However, in-depth analyses at the synaptic level by assessing morphology and function of purified preparations of nerve terminals, synaptosomes, and of astrocyte processes, gliosomes, prepared from the cerebral cortex of adult SMOX-overexpressing and control mice, revealed some differences. A lower content of Spm was found in the astrocyte processes of Dach-SMOX mice as compared to controls, while no difference was found in the PA content in the nerve terminals [43]. This finding is in some ways puzzling, because in Dach-SMOX mice, SMOX is specifically overexpressed in neuronal cells. Despite the overexpression of the Spm catabolic enzyme, PA levels were maintained in the nerve terminals, indicating homeostatic control of intracellular PAs at neuronal level. Astrocytes, in some ways, seem able to keep a constant level of Spm in neurons, in spite of the catalytic activity of SMOX. As already mentioned, PAs, such as Spm and Spd, are involved in glial-neuronal communication, are not synthesized, but are stored almost exclusively in glial cells [16], from which they can be released to regulate neuronal synaptic activity [44,45]. Organic cation transporters’ potential pathways allowing the transfer of PAs in and out of astrocytes [44,45,75,76]. The low level of Spm observed in astrocyte processes can be explained by hypothesizing that astrocytes are excreting Spm via connexins and/or organic cation transporters to replenish neurons depleted of Spm and keep a constant concentration of this molecule as well as the right Spm/Spd balance within neuronal cells. By considering that Spm can act as an internal cell blocker mediating rectification of Ca^2+^-permeable AMPA [19,20,77], as well as of inwardly rectifying Kir4.1 channels [17,78,79], reduced content of Spm in astrocyte process of Dach-SMOX mice could take part in the astrocyte processes dysfunction, possibly contributing to revert the astrocyte action from neuroprotective to detrimental.

## 5. Expression of AMPA GluA2-Lacking Receptors linked to Ca^2+^ Entry and Activation of Glutamate Release from Reactive Astrocyte Processes

The expression of the GluA1 subunit of the glutamate ionotropic receptor AMPA and of its phosphorylated form at serine 831 (Ser 831) site increased in astrocyte processes from Dach-SMOX mice [37]. The phosphorylation of GluA1Ser831 increases conductance of AMPA receptors, increasing neuron excitability and the strength of synaptic transmission in long-term potentiation [80,81]. Notably, an increase in the GluA1Ser831 and AMPA receptor localization at the cell membrane were reported to be ROS-dependent [82], and PKC was reported to be involved in AMPA receptor phosphorylation at Ser831 [83]; this is in line with increased expression of GluA1 and of the GluA1Ser831 subunit in astrocyte processes of Dach-SMOX mice, which suffer chronic oxidative stress with increased ROS production [12] and express higher levels of PKC [38]. Interestingly, GluA1 phosphorylation at Ser831, caused by early-life-induced seizures was related to seizure susceptibility in adult rodents, and the phosphorylation of GluA1Ser831 was increased in postmortem hippocampal samples from patients who had experienced neonatal seizures [81], suggesting that AMPA receptors and AMPA receptor phosphorylation at Ser831 might play a role in epileptogenesis [84]. Activation of AMPA receptors evoked Ca^2+^ influx in Dach-SMOX mice astrocyte processes [43] and glutamate release from the processes [36], while it was completely ineffective on both Ca^2+^ influx and glutamate release in the processes from control mice [36,43]. The lower Spm content in the processes is in line with functioning of these Ca^2+^-permeable GluA2-lacking AMPA receptors [19,20,77]. As a matter of fact, regulation of Ca^2+^ is of pivotal importance in the astrocyte functioning in CNS connectomics and is finely tuned in different subcellular regions of the astrocytes. Attention is now directed to the so-called Ca^2+^ microdomains, dynamic Ca^2+^ changes spatially restricted to fine PAPs [85,86]; the Ca^2+^ transients at the level of PAPs seems to depend mainly on Ca^2+^ influx, while the Ca^2+^ transients at astrocyte soma may depend on Ca^2+^ release from intracellular stores [85,87]. The Ca^2+^ influx mediated by Ca^2+^-permeable GluA2-lacking AMPA receptors in the astrocyte processes from SMOX mice indicates activation of a new channel receptor for Ca^2+^ in these processes, and its coupling to vesicular release of glutamate. The finding would open new investigation for a better understanding of Ca^2+^ microdomains in PAPs from reactive astrocytes.

## 6. Increased xc^−^ Transporter and Increased Glutamate In-Out Transport from Reactive Astrocyte Processes

The cystine-glutamate transporter xc^−^ is an amino acid antiporter expressed mainly on astroglial cells [88,89], which mediates internalization of cystine and in-out transport of glutamate. Dach-SMOX mice show increased expression of the astrocytic xc^−^ system transporter, in fact, cerebro-cortical slices from Dach-SMOX mice presented a higher expression of XCT (the catalytic subunit of xc^−^ system transporter) in comparison to controls, and in vivo kainate treatment induced an increase in XCT positive cells significantly higher in Dach-SMOX than in control mice [37]. The expression of this transporter is directly regulated by the nuclear factor erythroid 2-related factor 2 (Nrf2) [88,89], involved in antioxidant cellular defense, which was activated in Dach-SMOX mice after kainate treatment [37]. In Dach-SMOX astrocyte processes, a greater glutamate-releasing response to extracellular cystine as compared to controls was observed, consistent with higher expression of functional xc^−^ system transporters [37]. The activity of the xc^−^ system transporters and the consequent glutamate release response was inhibited by the transporter inhibitor sulfasalazine [37]. It can be surmised that the persistent oxidative stress in Dach-SMOX mice is responsible for the increased expression of these transporters and consequently of a higher glutamate release from astrocytes.

## 7. Reduced Expression of EAAT1 and EAAT2 in Reactive Astrocyte Processes

The excitatory amino acid transporter-1 (EAAT-1) and the excitatory amino acid transporter-2 (EAAT-2) are mainly localized on the plasma membrane of the astrocyte processes surrounding synapses and are the most important carriers involved in the clearance of glutamate and in keeping low extracellular levels of glutamate at the synapse level [90]. In kainate-treated Dach-SMOX mice, the expression of EAAT-1 was significantly more impaired than in controls [37]. The greater impairment of EAATs expression in excitotoxic conditions in Dach-SMOX mice is probably a consequence of chronic oxidative stress, as EAATs possess redox-sensitive elements that, once oxidized, can result in a decrease in EAATs expression and activity [91,92]. The lower expression of EAAT-1 in Dach-SMOX mice may be responsible for a reduced ability of the processes to clear glutamate from the synaptic cleft, therefore potentiating excitotoxicity. The processes of reactive astrocytes in Dach-SMOX mice may therefore sustain a positive loop; the release of glutamate from neurons might activate astrocytic AMPA receptors, evoking a further release of glutamate, while increased astrocytic glutamate release through the xc^−^ exchange, and reduced astrocytic glutamate uptake by EAATs, could participate in the loop further increasing extracellular glutamate and contributing to a chronic increase in neuronal excitability and excitotoxicity.

## 8. Neuron Loss

A significant decrease in the number of NeuN-positive cells in Dach-SMOX cerebral cortex was reported [12,36]. The neuron loss in Dach-SMOX mice is consistent with the pronounced brain damage, and with the higher number of neurons exhibiting cytoplasmic condensation and nuclear basophilia in the neocortex in response to in vivo kainate treatment [12]. The nerve terminals appear reduced in number as indicated by the reduction of synaptophysin-positive particles in the cerebral cortex of Dach-SMOX mice [36]. The nerve terminals maintained the ability to release glutamate in response to glutamatergic AMPA receptor activation [36], but at a closer functional analysis, they appear suffering, with a reduction of expression of the AMPA GluA1 subunit and of the subunit phosphorilated at Ser831 [37], a defective control of the intracellular Ca^2+^ response to AMPA receptor activation [43], and reduction of the antioxidant defense ability, as indicated by depletion of catalase [43]. In fact, the oxidative stress and derangement of glutamatergic transmission in neuron-glial networks might lead to an imbalance between neuroprotective and neuro-aggressive factors, reducing the neuron defense reserve and contributing to increased vulnerability to excitotoxic and oxidative damage. Moreover, as already mentioned, defective neuron function might lead to impairment of synaptic signaling from neurons to astrocytes [65], in turn affecting astrocyte-neuron communication. It can be hypothesized that impairment of synaptic signaling from neurons to astrocytes in Dach-SMOX mice might involve reduction of PA (Spm) release to replenish astrocytes; in turn, reduced Spm level into astrocytes would lead to a diminished ability of astrocytes to supply neurons with Spm during excitotoxic conditions [43,44,45], in a self-sustaining circle of deprivation of trophic factors. As outlined below, neuron suffering and damage are likely to be related to both chronic oxidative stress and chronic excitotoxic stress.

## 9. Chronic Oxidative Stress

Growing evidence supports the hypothesis that oxidative stress plays a role in different brain disorders [93,94]. In CNS, ROS are continuously produced in both physiological and pathological conditions, and CNS appears highly susceptible to ROS, which trigger lipid peroxidation, ultimately leading to neuron death [93]. The main enzymatic defense against ROS is provided by superoxide dismutase (SOD) and catalase, catalyzing dismutation of superoxide radicals and degrading H_2_O_2_, respectively. The non-enzymatic scavenger metallothioneins (MTs), up-regulated in neurodegenerative diseases [95], also provide protection against ROS. MT-1 and MT-2 are mainly expressed in astrocytes and behave as neuroprotectant against oxidative stress [96], while MT-3 is mainly expressed in neurons and seems to be involved in neuronal Zn^2+^ homeostasis [97]. Production of H_2_O_2_, generated as Spm oxidation product by overexpressed SMOX, was greatly enhanced in the brain cortex of Dach-SMOX mice [12], leading to chronic oxidative stress. In fact, 8-Oxo-2′-deoxyguanosine (8-oxo-dG), a major product of DNA oxidation and a marker for oxidative DNA damage [98], was highly expressed in the brain cortex of Dach-SMOX mice [37]. After in vivo kainate treatment, the number of 8-oxo-dG immunoreactive cells in the cerebral cortex increased in both SMOX-overexpressing and control mice, indicating that kainate is indirectly able to produce oxidative DNA damage; however, SMOX-overexpressing mice exhibited a stronger signal in both excitotoxic and basal conditions [37]. Furthermore, the transcription factor Nrf2, which plays a crucial role in cellular defense against oxidative insults [99], was activated in the Dach-SMOX cerebral cortex cells, as indicated by the increased ratio between nuclear and cytosolic Nrf2 [37]. It is worth mentioning that activated Nrf2 induces the expression of ARE genes including the xc^−^ system transporter as discussed above (Increased xc^−^ transporter and increased glutamate in-out transport from reactive astrocyte processes). Consistent with persistent oxidative stress, stimulation of SOD and catalase activities, and of MTs’ gene expression were described in the brain cortex of SMOX-overexpressing mice [36]. Remarkably, MT-1/MT-2 also reduce activation and recruitment of monocytes/macrophages and T cells and counteract microglial activation [100]; in fact, an increased number of microglial cells was observed in the cortex of the Dach-SMOX mice [12]. This antioxidant response was confirmed by a deeper analysis at the cerebrocortical synapses level of Dach-SMOX mice, which revealed the stimulation of catalase activity in astrocyte processes [43] and a reduction of catalase activity in the nerve terminals [43]. The catalase activity reduction in the nerve terminals might be a sign of neuron damage; in fact, catalase deficiency or malfunctioning was found to be associated with neurodegenerative disorders, such as Alzheimer’s and Parkinson’s disease, and neuron damage [101]. Taken together, the findings indicate that neurons and astrocytes in the cerebral cortex of Dach-SMOX mice undergo chronic oxidative stress condition. It can be surmised that in such a condition, chronic activation of defense mechanisms serves to maintain a balance between oxidants and antioxidants; as a consequence of even small oxidative insults, the antioxidant system could be overwhelmed, resulting in a shift toward oxidants and accumulated cell damage in time [102,103]. This chronic condition is to be distinguished from acute generations of the Spm oxidation products H_2_O_2_ and 3-AP in cerebral ischemia (or in acute H_2_O_2_ or 3-AP in vitro treatment), where the amount of agents would need to be higher to cause cytotoxicity [30,104]. Persistent activation of PA catabolism and consequent chronic imbalance of radical homeostasis might be better related to chronic pathological conditions involving oxidative mechanism activation, as compared to acute oxidative insults. Interestingly, an imbalance in the brain redox state, due to increased ROS production or failure of antioxidant systems, was described in neurodegenerative diseases [105].

## 10. Chronic Excitotoxic Stress

**Figure 5:**
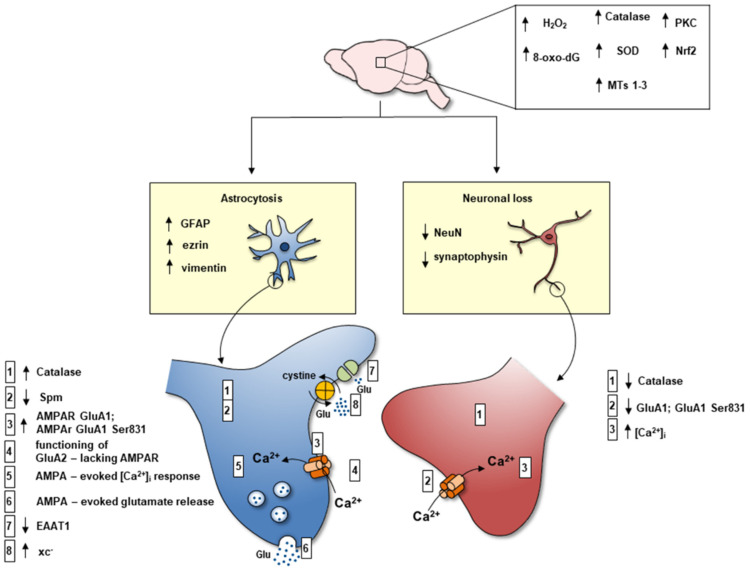
Schematic representation of the main molecular changes occurring at cerebrocortical glutamatergic synapses in the SMOX-overexpressing transgenic mouse model. The changes observed in the cerebral cortex are the increase in H_2_O_2_ production, 8-Oxo-2′-deoxyguanosine (8-oxo-dG), superoxide dis-mutase (SOD), catalase, metallothioneins (MTs), nuclear factor erythroid 2 related factor 2 (Nrf2), xc^−^ transporter, and PKC. Other changes consist in the relative abundance increase in astrocyte processes and decrease in nerve terminals (increase in GFAP, ezrin, and vimentin-positive cells versus reduction of synaptophysin and NeuN-positive cells). The main changes in astrocytes are: (1) increased level of catalase in astrocyte processes; (2) reduced Spm content; (3) increased expression of GluA1 and GluA1 phosphorylated at Ser 831 in astrocyte processes; (4) expression of functional GluA2-lacking Ca^2+^ -permeable AMPA receptors; (5) AMPA-evoked Ca^2+^ influx in astrocyte processes; (6) AMPA-evoked glutamate release from astrocyte processes; (7) lower expression of EAAT-1 (and EAAT2) after in vivo kainate treatment; (8) increased function of xc^−^ transporter (increased glutamate in-out transport). The main changes in neurons are: (1) depletion of catalase (reduction of the antioxidant defense ability in nerve terminals; (2) reduced expression of GluA1 and GluA1 phosphorylated at Ser 831; (3) defective control of the AMPA-evoked intracellular Ca^2+^ response in nerve terminals.

**Figure 6:**
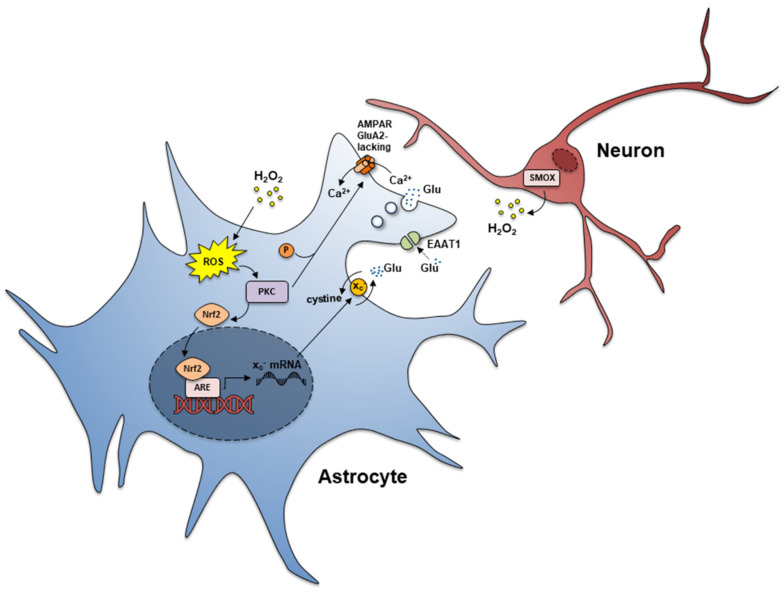
Possible mechanisms underlying dysfunctional pathways depending on chronic oxidative stress induced by SMOX overexpression. Neuronal SMOX overexpression is responsible for H_2_O_2_ increase leading to ROS augmentation within astrocytes. ROS increase induces PKC-Nrf2 cascade. The increase in PKC lead to higher phosphorylation of Glur1Ser831 of AMPAR, the phosphorylation and nuclear translocation of Nrf2. The transcription factor Nrf2 is the major regulator of phase II ARE genes. The nuclear translocation of Nrf2 induces a higher Xc- transporter expression, with a deregulated cystine import and glutamate export. ROS contribute to the reduction of EAATs and consequently a reduced glutamate uptake from the synaptic cleft. In the astrocytes of Dach-SMOX mice, the increase in Glur1Ser831 phosphorylated in the AMPA receptors contributes to the expression of functional GluA2-lacking AMPA receptors evoking Ca^2+^ influx and vesicular glutamate release.

Excitotoxic mechanism activation is a well-known pathway for neuron damage in several chronic and acute CNS disorders, the shift to excitotoxicity being largely controlled by astrocytes [106,107,108]. In Dach-SMOX mice cerebral cortex reactive astrocytes may cause an increase in the extracellular glutamate synaptic levels, therefore directly contributing to chronic neuronal excitotoxicity. As already outlined, the processes of the reactive astrocytes became capable of expressing GluA2-lacking AMPA receptors, which allow Ca^2+^ entry and activate the release of glutamate when responding to glutamate itself, therefore participating in a positive feedback loop [36,43]. Moreover, in Dach-SMOX mice, the astrocyte processes contributed to increased extracellular glutamate through the activation of the xc^−^ transporter [37]. A reduction of the expression of the excitatory amino acid transporters EAAT1 and EAAT2, selectively expressed in astrocytes and responsible for glutamate clearance from the synapse [90], was also found in Dach-SMOX mice after in vivo kainate treatment [37]. Therefore, at least three mechanisms may be responsible for astrocyte-dependent increase in extracellular glutamate at the synapses, potentially contributing to excitotoxic neuron insult: the functioning of glutamate-releasing AMPA receptors, the functioning of the xc^−^ transporter, and a likely impaired glutamate clearance by EAAT1 and EAAT2 transporters. Ca^2+^ dysregulation and impaired control of Ca^2+^ signals in the nerve terminals following glutamate AMPA receptor activation may also contribute to the vulnerability of neurons to excitotoxic insult; in fact, the ability to remove and buffer Ca^2+^ seems to be an important determinant of neuron susceptibility to excitotoxicity [109]. It is likely that the occurrence of excitotoxic and oxidative stress during the life of SMOX-overexpressing mice may lead to neuron suffering and ultimately to neuron loss. Reactive astrocyte processes may play detrimental effects being responsible for increased extracellular glutamate and excitotoxic mechanism activation, and possibly for a reduced supply of neuroprotective trophic factors. Alteration of the neuron–glial bi-directional communication by imbalance of neuronal PAs, suggests that endogenous PAs play roles in maintaining healthy intercellular neuron–astrocytes signaling and in neuroprotection. In fact, in the chronic activation of neuronal PA catabolism, neurons may undergo recurring astrocytic-dependent insults by potentially self-sustaining glutamate cascades, and by potentially self-sustaining deprivation of trophic factors. Therefore, SMOX overexpression in cerebrocortical neurons leads to a complex scenario of synapse dysregulation with astrocyte dysfunction and neuron suffering summarized in Figure 5 and Figure 6.

## 11. Susceptibility to Seizure

Dach-SMOX mice did not exhibit spontaneous seizure but did show an increased susceptibility to seizure. Notably, vulnerability to seizure was reported to be correlated with the development of a PA response [110]. Indeed, following kainate-induced seizures, the PA interconversion pathway was activated in limbic areas of adult rats, suggesting that the PA pathway may be involved in brain excitability and participate in the neuronal damage [111,112,113]. The Dach-SMOX mouse line is characterized by chronic increased levels of oxidative and excitotoxic stress; notably, both oxidative stress [114,115] and excitotoxic insults [107,108,116] are known to be related to epileptogenesis. This mouse genetic model is also characterized by reactive astrocytosis and astrocyte processes dysfunction; notably, astrocyte’s contribution to epileptogenesis is widely recognized [107,117,118,119,120]. Dach-SMOX mice exhibit increased susceptibility to chemical-induced seizures in two classical models of acute epilepsy, kainate-induced [12] and pentylenetetrazol-induced [38] epileptic seizures, evoked with two different chemical approaches: by direct activation of excitatory glutamate ionotropic receptors (kainate), and by pentylenetrazole impairment of the GABA-A receptors for GABA (gamma-aminobutyric acid), the main inhibitory neurotransmitter in CNS. In kainate-injected Dach-SMOX mice, a decrease in the Spm/Spd ratio was observed in the cerebral cortex, which could be responsible for higher kainate sensitivity [12]. It is conceivable that the increase in Spd level competes for Spm binding to AMPA and kainate receptors, enhancing the kainate effect on both receptor channels [17,121,122]. Increased susceptibility of Dach-SMOX mice to kainate seizure was confirmed by an in vitro approach [36]. An in vitro model of epileptic-like activity in combined hippocampus-neocortex slices, recorded with a multi-electrode array device, confirmed the increased susceptibility to kainate-evoked cortical epileptogenic activity in Dach-SMOX mice, and indicated that it was dependent on astrocyte function. In fact, spontaneous activity at low kainate concentration appearing only in slices from Dach-SMOX mice is consistent with the lower seizure threshold and increased sensitivity to kainate observed in vivo in kainate-injected Dach-SMOX mice [12]; the epileptogenic activity was dramatically reduced by fluoroacetate, a specific inhibitor of the astrocyte metabolism [36], indicating the involvement of astrocytes in the increased susceptibility to kainate-evoked seizures in the Dach-SMOX mice. Consistently, kainate activated AMPA receptors in astrocyte processes obtained from Dach-SMOX, but not from control, mice to evoke the release of glutamate, therefore potentially contributing to kainate-evoked seizures and increased susceptibility to epileptogenesis [36]. As already mentioned, PKC activation and AMPA receptor phosphorylation at Ser831 has been suggested to play a role in epileptogenesis [81,83,84]; notably, an increase in PKC expression was reported in Dach-SMOX mice cerebral cortex [38], and an increase in AMPA receptor phosphorylation at Ser831 was reported in astrocyte processes [36]. Also, Ca^2+^-permeable AMPA receptors have been identified as contributing to excitotoxicity in epilepsy in animal models, although human data would suggest that the involvement of Ca^2+^-permeable AMPA receptors in epilepsy depends on the stage of disease and the age of subjects [108]. Furthermore, inhibition of the xc^−^ system by sulfasalazine treatment, which, in vitro, inhibited the increased sensitivity of the astrocytic xc^−^ transporter to cystine, obliterated the increased susceptibility of SMOX mice to epileptogenic activity evoked in vivo by kainate, restoring an epileptic response comparable to the one of control mice [37]. In Dach-SMOX mice, changes of reactive astrocyte processes and their contribution to increase the extracellular glutamate levels may be important to determine a chronic increase in neuronal excitability. Indeed, neuronal hyperexcitability and high levels of extra-synaptic glutamate is known to be linked to epileptogenic activity [123]. Furthermore, alteration of Kir4.1 channel function and of the regulation of extracellular K^+^ is also expected, as a consequence of both reduced Spm content and of reactive astrocytosis. Indeed, Spm decreased inward rectification in Kir4.1 channels [78,79,124] contributing to the hyperpolarized membrane potentials required for K^+^ spatial buffering [125]. Notably, defective activities of glial Kir4.1 was shown to impair K^+^ clearance following synaptic activity [126] and evoke epileptic activity [127]. In conditions of enhanced neuron activity and impaired K^+^ clearance, accumulation of extra-cellular K^+^ may trigger the onset of epileptic seizures [125,128]. In fact, involvement of astrocytic Kir4.1 channel dysfunction [129] and the contribution of impaired astrocytic K^+^ regulation [130] to epileptogenesis are increasingly recognized.

## 12. Conclusions

In conclusion, the SMOX-overexpressing mouse model Dach-SMOX, with chronic astrocyte dysfunction, oxidative stress, and dysregulation of glutamatergic transmission (Figure 5 and Figure 6), could help in shedding lights on the susceptibility to epileptic seizures, possibly contributing to understanding the mechanisms underlying epileptogenesis in vulnerable individuals.

Furthermore, the genetic model of Dach-SMOX mice could provide new molecular mechanism targets to search for novel antiepileptic drugs. Indeed, awareness of the limitations of acute seizure models has increased in the last decades. In fact, the complexity of epileptogenesis mechanisms likely reflect a disturbed network of interactions and targeting more than one mechanism is proposed for developing anti-epileptogenic and disease-modifying new therapies [131]. Strategies for antiepileptic drug discovery are moving to new models focusing on molecular mechanisms underlying susceptibility to epilepsy, to reveal targets contributing to epileptogenesis and identify therapies interfering with the processes in prone individuals [131,132]. These models can reproduce more illness characteristics of the epilepsy complexity than exclusive use of simple acute seizure models, particularly when testing target-based strategies for drug development in drug-resistant epilepsy or against seizure susceptibility [131,133,134]. In fact, new perspectives for design and discovery of antiepileptic drugs are focusing on astroglial modulation of spatiotemporal seizure dynamics [118,119,120] Notably, the SMOX-overexpressing mouse model recapitulates the conditions, namely excitotoxic mechanism, oxidative stress, and reactive astrocyte participation, that, by general consensus, have been linked to epileptogenesis [107,108,115,119,120]. All these conditions depend specifically on chronic activation of PAs’ catabolism in the Dach-SMOX mouse line.

It is of relevance here to underline that natural and synthetic PAs were reported to exhibit anti-convulsant properties and neuroprotective roles in models of epilepsy, including non-mammalian models [135,136]. Indeed, neuroprotective effects of Spm following hypoxic-ischemic-induced neuron damage was reported in vitro [137] and confirmed in vivo in rat pups [138]. In a seizure model in *Xenopous laevis*, activity-dependent increased synthesis of Put and had a protective effect to a second convulsant exposure, suggesting a neuroprotective role for PAs in the developing brain [135]. Also, in rodents, Put overproduction was found to be related to an elevated seizure threshold to both chemical (pentylenetetrazol) and physical (electroshock) stimuli [139]. Putrescine was also reported to protect against kindling-evoked seizures [140,141]. Furthermore, and interestingly, the synthetic polyamine Mygalin was effective as an anticonvulsant in a rodent model of chemically induced seizures [136].

## Data Availability

Data available upon request from the corresponding authors.
